# Sexual and reproductive health services utilization and associated factors among adolescents attending secondary schools

**DOI:** 10.1186/s12978-022-01468-w

**Published:** 2022-07-15

**Authors:** Chaltu Abdurahman, Lemessa Oljira, Saba Hailu, Melkamu Merid Mengesha

**Affiliations:** 1 College of Health and Medical Sciences, Dire Dawa University, Dire Dawa, Ethiopia; 2 School of Public Health, College of Health and Medical Sciences, Haramaya University, Harar, Ethiopia; 3 School of Public Health, College of Medicine and Health Sciences, Arba Minch University, Arba Minch, Ethiopia

**Keywords:** Sexual and reproductive health, Adolescent, Service utilization

## Abstract

**Background:**

Sexual and reproductive health (SRH) is referring to physical and emotional wellbeing and includes the ability to be free from unwanted pregnancy, unsafe abortion, sexually transmitted infections including HIV/AIDS, and all forms of sexual violence and coercion. SRH is the main services packages that prevent and reduce adolescent reproductive health risks and problems. While the government of Ethiopia has undertaken several measures to improve SRH services, there was limited data on utilization among adolescents and associated factors.

**Objective:**

To assess utilization of SRH services and associated factors among adolescents attending secondary schools in Haramaya District, Eastern Ethiopia.

**Methods:**

A **s**chool-based cross-sectional study was conducted among adolescent students aged 15–19 years. A total of 692 adolescents were selected using a multi-stage sampling from two randomly selected secondary schools, each from rural and urban settings, in Haramaya district where 642 provided complete data and included in the analysis. A structured, pretested, and self-administered questionnaire was used to collect data. Data entry was conducted using Epi Data version 3.1 and exported to STATA version 16 for analysis. Bivariable and multivariable binary logistic regression were used to identify factors associated with school adolescents’ utilization of SRH. Statistically significant associations are declared at P-value < 0.05.

**Result:**

A total of 642 completed the survey questionnaire, constituting a response rate of 92.7% (642/692). Male adolescents accounted 63.7% and the mean age of respondents was 17.71 years. Among those who completed the survey, 23.5% (95% CI: 20–26.8) utilized SRH services. Adolescents who were exposed to SRH information (adjusted odds ratio (AOR) = 2.11, 95% CI: 1.22–3.6), aware of SRH service providing facility (AOR = 1.83, 95% CI: 1.12–3.0) and SRH service components (AOR = 2.76, 95%, CI: 1.53–4.97), and distance from SRH facilities (AOR = 2.28, 95%, CI: 1.13–4.62) were significantly associated with the utilization of SRH services.

**Conclusion:**

Nearly one-in-four secondary school adolescents (23.5%) utilized SRH services. Targeted promotion of SRH providing facilities and SRH service components aimed at awareness creation could improve adolescents’ utilization of SRH services. Improved SRH services utilization among adolescents who were far from SRH services providing facilities needs further investigation.

## Plain language summary

**Background:**

Sexual and reproductive health (SRH) refers to physical and emotional wellbeing and includes the ability to be free from unwanted pregnancy, unsafe abortion, sexually transmitted infections including HIV/AIDS, and all forms of sexual violence and coercion. Components of the SRH services are important to reduce adolescent reproductive health risks and problems. This paper reports on the frequency of in-school adolescents who utilized SRH services and the factors that determined it.

**Methods:**

we collected data from 642 in-school secondary school adolescents, aged 15–19 years, in Haramaya district, East Ethiopia. The data were collected from two schools, one from rural and the other from urban areas, using lottery method from the available schools in the district. A face-to-face interview was conducted to obtain data from the adolescents in the selected secondary schools.

**Result:**

Out of 642 in-school adolescents, 23.5% utilized SRH services in Haramaya District. Adolescents who previously received SRH information, who knew SRH services providing facility, aware of SRH service components, and those who live not close to the SRH facilities were utilized the SRH services more often compared to their friends.

**Conclusion:**

Nearly one-in-four secondary school adolescents (23.5%) utilized SRH services. Targeted promotion of SRH providing facilities and SRH service components aimed at awareness creation could improve adolescents’ utilization of SRH services.

## Introduction

Sexual and reproductive health (SRH) refers to physical and emotional wellbeing and includes the ability free from unwanted pregnancy, unsafe abortion, STIs including HIV, and all forms of sexual violence and coercion. The majority of people become sexually active during the adolescence period putting people in this age group at higher risks of SRH problems [1]. The World Health Organization (WHO) defines an adolescent as an individual aged from 10 to 19 years [2]. Adolescence is a transitional phase of growth and development during which of the most rapid and complex life stages characterized by a significant physical, cognitive, behavioural, social, and psychological change occurs [3]. This development was characterized by suboptimal decisions and actions that put them to unintentional injury and risk-taking [4].

Globally about 1.2 billion of the total population are adolescents and more than half of this population lives in developing countries [5]. Sub-Saharan Africa (SSA) is the region where 23% of its total population (1.06 billion) were adolescents [6]. In Ethiopia, one of a country in the SSA with a rapidly growing population of adolescents and youths, adolescents account for 33.8% of the estimated total population of 90 million [7].

Pregnancy, abortion, and STIs (including HIV) are the major SRH risks that result in adverse reproductive health outcomes in adolescents than adults. According to the 2018 Inter-Agency Working Group on Reproductive Health (IAWG) report, AIDS-related death among adolescents are nearly tripled from 21,000 in 2000 to 60,000 in 2014, one-in-four women give birth during adolescence or before they reach their 18^th^ birthday, and 3.9 million females aged 15–19 years undergo unsafe abortion every year [5]. A national-level report of the Ethiopia Demographic Health Survey in 2016 indicated that the adolescent fertility rate was 80 births per 1000 [7]. Female adolescents aged 15–19 years were seven times more likely to be HIV positive than male adolescents [8]. Research evidence reveal that early pregnancy, abortion, and STIs (including HIV infection) rates are high among adolescents, and hence adolescent and youth reproductive health is a public health concern [9].

Sexual and reproductive health services include access to information and services on prevention, diagnosis, counselling, treatment, and care, and require that all people can safely reach services without traveling for a long time or distance [10].

A study conducted in higher education institutions in Ethiopia revealed that one-third of university students have already been sexually experienced. Of these, nearly two-thirds were found to have sexual experience already before joining higher education institutions evidencing that SRH problems manifest early on and calls for interventions at early adolescence [11]. A range of social norms and practices that prevent sexually active adolescents from accessing contraceptives, maternity care, and other services due to their age and gender is challenging for effective service delivery [9, 12]. A qualitative study by Munea et al. in Northwest Ethiopia reported that the community was intolerant to adolescent premarital sex and did not approve neither SRH use nor SRH communication with unmarried adolescents[13]. Consequently, the main barriers preventing adolescents to access SRH services was related to cognitive accessibility (a lack of sexual knowledge and a lack of awareness of services) and psychosocial accessibility (feelings of shyness and shame, fear of parents finding out service use, and lack of confidentiality) as noted by Thongmixay et al. [14]. Low service uptake due to these challenges were affecting adolescent health and it compromise the educational attainment of adolescents, increases dependency, and reduce the economic potential of the country [15].

The International Conference on Population and Development (ICPD) in 1994 held in Cairo established a comprehensive SRH service that needs to address and solve the SRH problem for adolescents and youth [16]. National strategy for adolescent and young sexual and reproductive (AYSR) health aims to increase access to information, education and to promote health service uptakes by adolescents and youth [8].

Despite that utilization of ASRH are not well explored in Ethiopia, previous studies focused on youth and adult people. A few studies that assessed ASRH services, in turn, are limited to selected service components[17]. Therefore, this study aimed to assess the utilization of SRH services among secondary school adolescent students in Haramaya District, Eastern Ethiopia.

## Materials and methods

### Study design and settings

We conducted a school-based cross-sectional study in Haramaya District which is located at 500 km away from Addis Ababa, the capital of Ethiopia. When this study was conducted, the district has a total population of 313,152 (50.9% were males). Only 16.9% of the total population were urban residents with the significant majority residing in the rural parts of the district. There are eight health centers and one general hospital in the study area. The district has 6 secondary schools (3 in urban and 3 in rural areas) and enrolled a total of 6972 students in the 2019/2020 academic year (where 73.0% were males).

This study was conducted from March 1 to 15, 2020 in secondary schools at Haramaya District, East Hararge Zone, Oromia Regional State, Ethiopia.

### Sample size and sampling procedure

The sample size was calculated using a single proportion formula with the assumption of 95% confidence interval, 21.2% proportion of SRH service utilization [18] from the study conducted in Nekemte town, Western Ethiopia, 4% margin of error, 1.5 of design effect, and 15% of non-response rate was added the sample size resulting in a minimum sample size of 692. The final sample size considered for analysis was 642 due to consideration of completeness of data.

A multi-stage sampling method was applied to select representative sample of adolescent students. In the first stage, secondary schools in the Haramaya district were clustered into two groups (urban and rural), then from each cluster one school was selected by a simple random sampling method, making a total of two schools. Secondly, school adolescents were stratified by their grade level of attendance. Sample size was then allocated proportionally after obtaining list of students from the respective school administration. Finally, the study participants were selected from each grade by using simple random sampling from the sampling frame of student roster.

## Outcome variable

The dependent variable of interest in this study was adolescent SRH service utilization. The definition of the utilization of the SRH services was ever utilization of any one of the following SRH services:

- SRH information Education and counselling
- Contraceptive service
- Pregnancy test & care
- Voluntary counselling and testing (VCT)
- Sexually transmitted infections (STI) screening, diagnosis, and management services
- Safe abortion care

The outcome variable was measured based on self-report using a “yes or no” response to a single question on whether participants have ever utilized any one or many of the specific SRH service components in private and government health facilities.

### Data collection tool and procedure

Data were collected using a pre-tested, self-administered questionnaire adapted from the John Cleland’s illustrative questioner for interviewing young people and reviewing works of literature. The tool consisted of three sections: socio-demographic, individual, and health facility characteristics. The original questionnaire was developed in English and then translated into the local Afan-Oromo language. The translated version was then translated back to the original English version for consistency. Six data collectors who were diploma graduates and two supervisors (BSc degree) conducted the data collection following orientation on study objectives, benefits of the study, research ethics and informed consent, and data collection techniques. Before the actual data collection, a pre-test was conducted to identify problems with the assessment tool in a small sample of students, 35(5%), outside the selected study setting. During the data collection, the principal investigator and the supervisors made daily checks to assess completeness of each returned questionnaire and questionnaires with several missing values for important variables have been discarded.

## Operational definition

### SRH service utilization

Refers to ever utilization of one or more of SRH service components in private and government health facilities [18]. Positive response to SRH service utilization was further validated by at least one type of services mentioned by respondents and those who received one or more service components were considered to have utilized SRH services. SRH information, education and counselling, contraceptive, pregnancy test & care, VCT, and HIV test, STI diagnosis and management, and Safe abortion care were the service components assessed in this study.

### Accessibility of RHS facility (geographical accessibility)

In this study, accessibility was measured based on adolescent’s self-report of distance they had to travel to health facilities providing SRH services.

## Data processing and analysis

Data were entered into Epidat version 3.1 and then exported to STATA version 14 for statistical analysis. Descriptive results were presented in tables, figures, and using numerical summary measures for continuous variable. To explore relationship between variables, cross-tabulation was conducted for variables of interest against the outcome variable. SRH services utilization was measured using a dichotomous response (Yes = 1 and No = 0). The positive response was further validated with questions on the type of SRH services utilized. The proportion of SRH service utilization was computed with specific service components. Family wealth index was computed using principal component analysis (PCA) by considering locally available household assets and categorized in to five (very poor, poor, medium, rich and very rich). Bivariable binary logistic analysis was conducted to select candidate variables for the multivariable binary logistic regression. Variables with P-value less than 0.25 in bivariable binary logistic regression model proceeded into the multivariable binary logistic regression model. Moreover, model fitness was checked by the Hosmer–lemeshow test and multi-collinearity of variables in the final model was also checked using the variance inflation factor (VIF). Variables in the final model with P-value < 0.05 were considered as statistically significant predictor of SRH service utilization. Effect estimates were reported using odd ratio with a corresponding 95% confidence interval.

## Result

### Socio-demographic characteristics of participants

A total of 642 adolescent students have participated in the study which makes a response rate of 92.7%. From the total respondents, 63.7% respondents were male students with the mean age of 17.71 years SD (± 1.19) and more than half, 57.6%, were grade 9 and 10. From the total participants 75.1% were single and 86.1% were Muslims. Regarding the living arrangement of students, most of them, 81.5%, were living with their families (Table 1).

**Table 1:** Socio-demographic characteristics of secondary school adolescents who participated in sexual and reproductive health service utilizations study in 2020, Haramaya district, Ethiopia

Characteristics	Category	Frequency	Percentage %
Sex	Male Female	409 233	63.7 36.3
Age group	15–17 18–19	231 411	36.0 64.0
Educational level	Grade 9^th^ Grade 10^th^ Grade 11^th^ Grade 12^th^	198 172 158 114	30.8 26.8 24.6 17.8
Marital status	Single In relationship Married	482 67 93	75.0 10.0 14.5
Religion	Muslim Orthodox Other	553 60 29	86.1 9.4 4.5
Ethnicity	Oromo Amhara Other	573 38 29	89.6 5.9 4.5
Mother’s Education	No formal education Elementary school Secondary school College and above	368 161 76 37	57.3 25.1 11.8 5.8
Father’s Education	No formal education Elementary school Secondary school College and above	318 117 115 92	49.5 18.2 18.0 14.3
Parent Residence	Rural Urban	378 264	58.9 41.1
Living arrangements	Living with family Not living with a parent	523 119	81.5 18.5
Own Income	Yes No	90 552	14.0 86.0
Family wealth quintile	Very poor Poor Medium Rich Very rich	129 128 129 128 128	20.1 19.9 20.1 19.9 19.9

### Secondary school adolescents’ characteristics

Of the total adolescents participated in the study 39% ever had a boy or girlfriend and 20% of were sexually active. 68.5% of participating adolescents reported that had been exposed to SRH information with 52% mentioned peers as the main source of information. Regarding discussion of SRH issues 62.2% respondents ever discussed at least two SRH issues: 42.6% discussed with peers followed by 19.8% discussed with healthcare providers (Table 2).

**Table 2:** Secondary school adolescents’ characteristics and exposure to sexual and reproductive health information in 2020, Haramaya district, Ethiopia

Variables	Category	Frequency	Percentage %
Boy/Girlfriend	Yes No	250 392	39.0 61.0
Ever had sexual intercourse	Yes No	132 510	20.6 79.4
Exposed for SRH information	Yes No	440 202	68.5 31.5
Source of SRH information (n = 440)	Parent Peers Teachers Health providers Others	39 229 122 43 7	8.9 52.0 27.7 9.8 1.6
Ever discussion about SRH issues	Yes No	399 243	62.2 37.8
Discussed SRH issues with (n = 399)	Parent Peers Health providers Boy/girlfriend Teachers	45 170 79 56 49	11.3 42.6 19.8 14.0 12.0
Perceived risks for SRH problems	Yes No	294 348	45.8 54.2
Information club in the schools	Yes No	230 412	35.8 64.2
Distance from SRH facility	Short distance Medium Very far	99 314 229	15.0 48.9 35.7

From all respondents, more than half of 56.1% knew health facilities that provide SRH services and 66.7% knew SRH service types. Out of these respondents, 90.9% knew VCT services, 73.1% knew contraceptive services and 29.0% knew safe abortion services (Table 3).

**Table 3:** Sexual and reproductive health service availability awareness among secondary school adolescents in 2020, Haramaya district in, Ethiopia

SRH facility and service	Category	Frequency	Percentage %
Know SRH facility whereabouts	Yes No	360 282	56.1 43.9
Source of information(n = 360)	Family Friends Teachers Media Notice board	5 196 22 69 68	1.4 54.4 6.1 19.2 18.9
Know SRH service types	Yes No	428 214	66.7 33.3
Types of SRH services aware of (n = 428)			
Contraceptive	Yes No	313 115	73.1 26.9
VCT service	Yes No	389 39	90.9 9.1
STI diagnosis and treatment	Yes No	291 137	68.0 32.0
Counselling and information service	Yes No	258 170	60.3 39.7
Pregnancy test and care service	Yes No	187 241	43.7 56.3
Safe abortion service	Yes No	124 304	29.0 71.0

### Utilization of SRH services

**Fig. 1:**
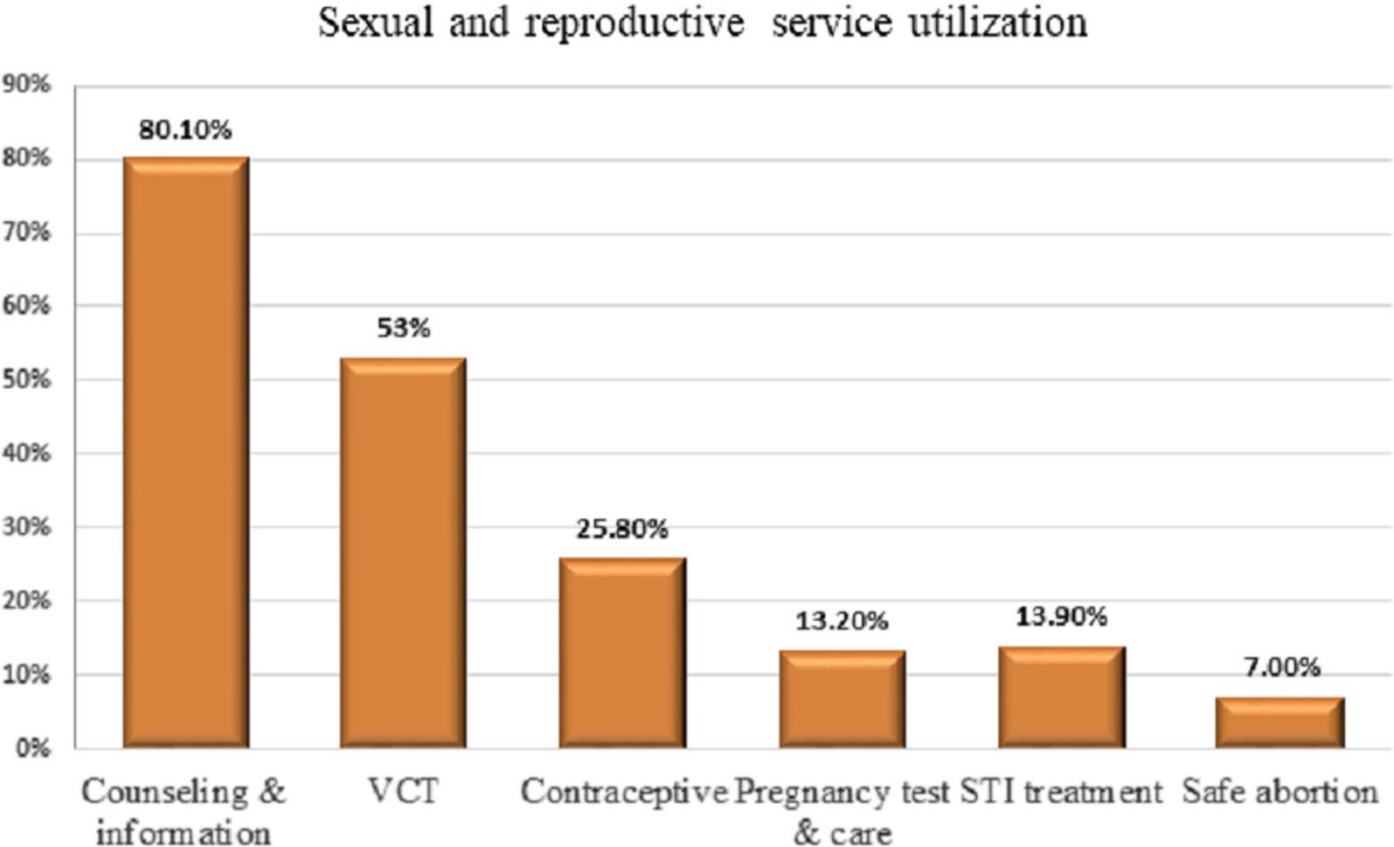
Utilization of SRH service components among in-school adolescents in 2020, Haramaya District, Ethiopia

**Fig. 2:**
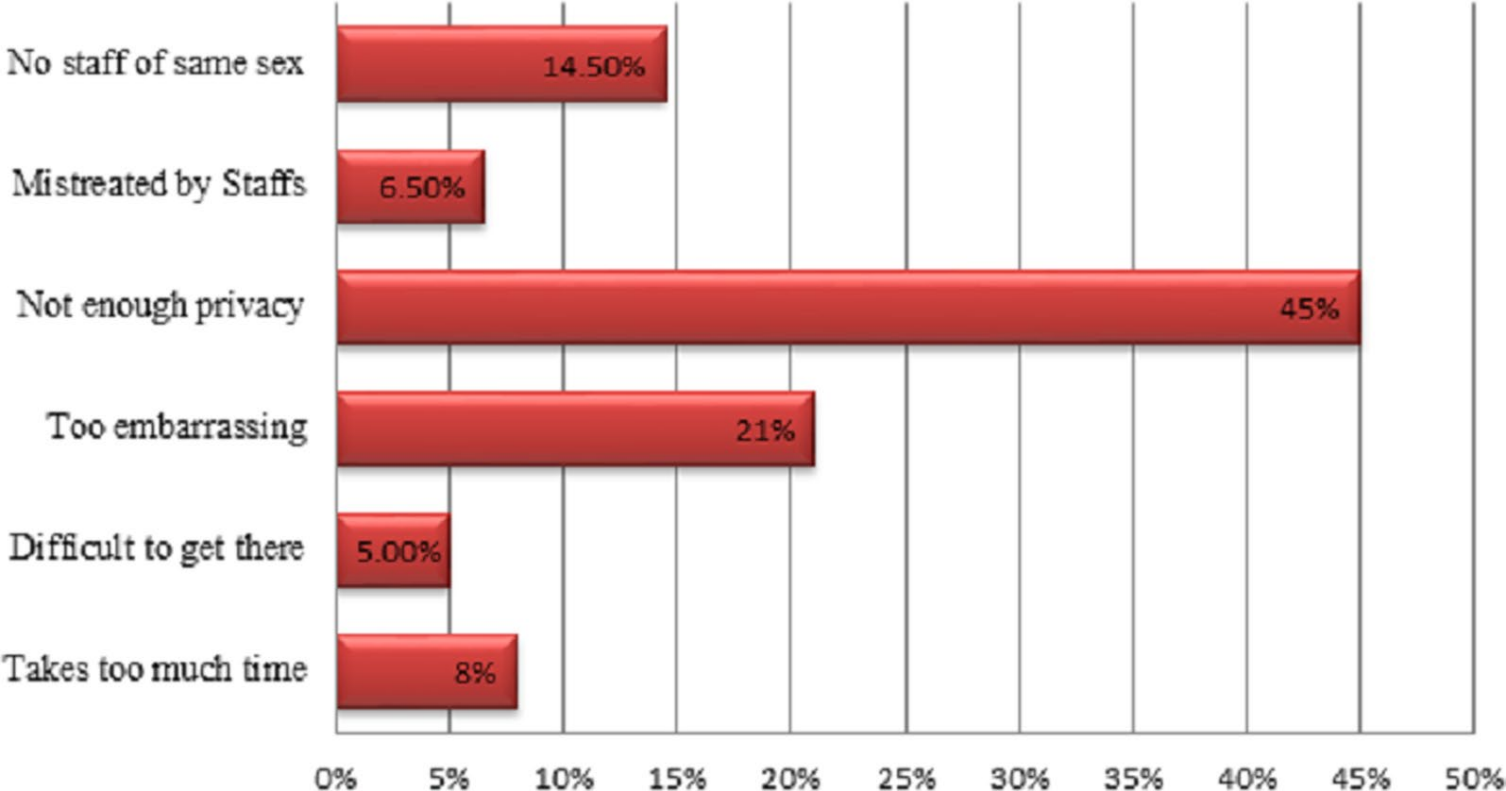
Main reasons for in-school adolescents not returning to Sexual and Reproductive Health facility in 2020, Haramaya District, Ethiopia

Overall, 23.5% (95% CI: 20, 26.8) of in-school adolescents utilized SRH services (64.2% male and 35.8% female students) in Haramaya district. The most frequently used SRH services components were counseling, information, and education, followed by VCT & HIV testing (Fig. 1). Of 132 sexually experienced adolescents, 42.4% used SRH services, of which 46.2% were married. Among adolescents who utilized SRH services, 41% did not want to return to the health facility. The main reasons for not wanting to return to health facilities were: not having enough privacy 28(45%), too embracing 13(21%), and no healthcare provider of same sex (14.5%) (Fig. 2).

### Factors associated with utilization of SRH

A binary logistic regression model was used to identify factors associated with utilization of SRH services. Based on the estimates from the bivariable binary logistic regression model, the factors significantly associated with utilization of SRH were age, marital status, living arrangement, having own income, ever had sexual intercourse, ever exposed to SRH information, discussion of SRH issues, perceived SRH risks, know of RH facility, aware of RH services, and being far at distant from the facility. All variables that showed association in the bivariable model are entered into the multivariable binary logistic regression model. Controlling for the effect of other variables in the model, adolescents with the following characteristics were over two-fold more likely to use SRH services: exposure for information (AOR = 2.11, 95% CI: 1.22–3.66), awareness of SRH services(AOR = 2.76 95%, CI: 1.53–4.97), identifying SRH facility whereabouts (AOR = 1.83 95% CI: 1.12–3.0), and far from SRH clinics (AOR = 2.28 95%, CI: (1.13–4.62) showed statistically significant association at p-value < 0.05 (Table 4).

**Table 4:** Bivariate and multivariate binary logistic regression analysis SRH service utilization among secondary school students in Haramaya District, 2020

Variables	Utilization of SRH		COR,95%CI	AOR.95%CI
	Yes (%)	No (%)		
Age				
15–17	40 (17.3%)	191 (82.7%)	1.00	1.00
18–19	111 (27%)	300 (73%)	1.77 (1.17–2.64)	1.23 (0.78–1.96)
Marital status				
Single	108 (19.7%)	441 (80.3%)	1.00	1.00
Married	43 (46.2%)	50 (53.8%)	3.51 (2.21–5.55)	1.37 (0.37–5.04)
Living arrangements				
With family	102 (19.5%)	421 (80.5%)	0.35 (0.23–0.53)	0.62 (0.22–1.74)
Not with family	49 (41.2%)	70 (58.8%)	1.00	1.00
Have own income				
Yes	33 (36.7%)	57 (63%)	2.13 (1.32–3.4)	1.47 (0.85–2.53)
No	118 (21.4%)	434 (76.6%)	1.00	1.00
Ever had sexual intercourse				
Yes	56 (42.4%)	76 (57.6%)	3.32 (2.13–4.85)	1.32 (0.61–2.81)
No	95 (18.6%)	415 (81.4%)	1.00	1.00
Exposed for SRH information				
Yes	130 (29.5%)	310 (70.5%)	3.62 (2.2–5.93)	2.11 (1.22–3.66)
No	21 (10.4%)	181 (89.6%)	1.00	1.00
Discussion of SRH issues				
Yes	118 (29.6%)	281 (70.4%)	2.67 (1.74–4.08)	1.13 (0.69–1.87)
No	33 (13.6%)	210 (86.4%)	1.00	1.00
Perception for SRH risk				
Yes	94 (32%)	200 (68%)	2.39 (1.64–3.49)	1.4 (0.91–2.16)
No	57 (16.4%)	291 (83.6%)	1.00	1.00
Awareness of SRH facility				
Yes	118 (32.8%)	242 (67.2%)	3.67 (2.4–5.62)	1.83 (1.12–3.0)
No	33 (11.7%)	249 (88.3%)	1.00	1.00
Awareness of SRH services				
No	18 (8.4%)	196 (91.6%)	1.00	1.00
Yes	133 (31.1%)	295 (68.9%)	4.9 (2.9–8.29)	2.76 (1.53–4.97)
Distance from Health facility				
Short walking	13 (13%)	86 (87%)	1.00	1.00
Medium	69 (22%)	245 (78%)	1.86 (0.98–3.53)	1.63 (0.81–3.24)
Very far	69 (30.1%)	160 (69.9%)	2.85 (1.49–5.4 5)	2.28 (1.13–4.62)

## Discussion

This cross sectional study assessed level of in-school adolescents’ SRH services utilization in Harmaya district, east Ethiopia, and identified factors associated with service utilization. The level of SRH services utilization was estimated at 23.5% (95% CI: 20.4, 27.0). Factors significantly associated with the in-school adolescents’ utilization of SRH services include exposure to SRH information, awareness of SRH services providing facility and service components, and distance from service providing facilities.

The level of SRH services utilization among in-school adolescents in the study setting was 23.5%. This finding was consistent with similar studies among in-school adolescents in Ethiopia and elsewhere where SRH services utilization reports ranged from 21.2 to 24.6% [18–21]. However, compared to other similar studies in Ethiopia, magnitude of the SRH services utilization observed in the study setting was low. For example, a study in southern Ethiopia reported an SRH services utilization of 33.8% among high school adolescents in youth friendly service implemented areas [22]. Similarly, a study in Debre Tabor town among high-school- and preparatory-students reported an SRH service utilization of 28.8% [23]. The SRH services utilization in the study setting, however, was high compared to adolescents in the general population (8.6%) [24] and those who reside in areas where youth friendly services are not implemented (9.9%) [22]. Part of the variation could be explained by differences in terms of factors related to service delivery like friendliness, negative attitude from service providers and feasibility of service hours; factors related adolescents’ perception (fear of being seen while using SRH services); and socio-cultural barriers [21, 25].

The most utilized SRH services components include information, education, and counseling (80.1%) where previous studies also reported a similar services utilization pattern that was largely limited to information seeking and counseling services [26]. The percentage of utilization of other services including contraception (28.5%), STI treatment (13.9%), pregnancy testing (13.2%), and safe abortion care (7%) were also comparable with previous studies [26]. However, this study did not assess adolescent’s unmet need of important services which might have shed light on the potential gaps to be addressed pointing to the services that adolescents seek but did not use.

Adolescents who previously had exposure to SRH information were significantly associated with current service utilization. Previous studies also reported consistent reports that adolescents who had multiple sources of SRH information including discussion with family members, friends, healthcare providers, and schools were more likely to use SRH services [22, 27, 28] as these may improve adolescents’ awareness on SRH related issues.

Adolescents who were aware of the specific SRH service components were more likely to utilize the services than those who did not know. Due to the nature of the study, however, it was not possible to state whether awareness of the specific SRH services components had led to service utilization or the reported awareness was secondary to the service utilization itself. Previous similar studies elsewhere emphasized the importance of adolescents’ awareness of SRH services providing facilities, particularly for rural adolescents [29]. It was further emphasized that lack of awareness of SRH service components and knowledge about youth friendly services among adolescents and where to go to use those services were the main reason for the low uptake of adolescent SRH services [29, 30].

Interestingly, in this study, adolescents who were far from health facilities that provide SRH services were two times more likely to utilize SRH services than those who lived close to SRH services providing facilities. This finding was against the reports in previous studies that indicated availability of service providing facilities in close by facilitated SRH services utilization [17, 19]. A systematic review by Nisiima et al. reported that facilitators of utilization of SRH services were mostly structural in nature including the quality of health services and clinics for adolescents to fit their needs and preferences [30]. Due to reasons related to confidentiality, availability of needed service, attitude of service providers, and shyness [17, 21], adolescents may prefer to use services from facilities where they could not be easily identified and find services of their preference. Poor understanding of the adolescents’ reproductive health needs in the community due to socio-cultural norms (not approving sexuality discussion during adolescents and hence utilization of services) and fear of adolescents to be seen by community members are big challenges impeding SRH use that need critical attention. In this study, for 58.9% of adolescents, their parents lived in rural areas and 18.5% of the adolescents lived alone in order to attend their high school study in the town of the Haramaya district. Consequently, the perceived freedom of living alone and confidentiality may have contributed to the observed association of distance and SRH services utilization.

Our study was not without limitation. Pertaining to sensitive nature of the topic studied, respondents may not give honest responses as required. This might underestimate the prevalence of SRH service utilization. We have tried to reduce issues related to confidentiality by making the data collection anonymous and using a self-administered structured questionnaire in the local language for data collection obtain information. This study cannot indicate the direction of the causal relationship.

## Conclusion

The level of in-school adolescents SRH services utilization was not satisfactory to prevent potentially amenable problems related to and associated impacts of sexual activity and pregnancy related outcomes of adolescents. Targeted promotion of SRH providing facilities and SRH service components aimed at awareness creation could improve adolescents’ utilization of SRH services. Improved SRH services utilization among adolescents who were far from SRH services providing facilities needs further investigation.

## Data Availability

All data pertaining to the findings are presented in this paper. However, the data can be obtained from the corresponding author any time on reasonable request.

## Abbreviations

AIDS: Acquired Immune Deficiency Syndromes
ASRH: Adolescent sexual and reproductive health
AOR: Adjusted odd ratio
CSA: Central statistic authority
EDHS: Ethiopian Demographic and Health Survey
FMOH: Federal Ministry of Health
FP: Family planning
HIV: Human immune virus
HTTP: Harmful traditional practice
IAWG: Inter-agency working groups
SRH: Sexual and reproductive health
SSA: Sub-Saharan Africa
STI: Sexual transmitted infection
UNFPA: United Nation Fund for Population Activities
UNICEF: United Nation International Children Emergency Fund
VCT: Voluntary counselling and testing
